# Potent anthelmintic activity of a colloidal nano-silver formulation (Silversol^®^) against the model worm *Caenorhabditis elegans*

**DOI:** 10.1186/s13104-023-06392-1

**Published:** 2023-07-03

**Authors:** Gemini Gajera, Chhaya Godse, Anselm DeSouza, Dilip Mehta, Vijay Kothari

**Affiliations:** 1 Institute of Science, Nirma University, Ahmedabad, 382481 India; 2 Viridis BioPharma Pvt. Ltd, Mumbai, India

**Keywords:** Nematicide, Anthelmintic, Colloidal Nano-silver, *Caenorhabditis elegans*

## Abstract

**Objective:**

In the background of a very small number of effective anthelmintics available today with a narrow activity spectrum, and a rise in resistance against them among parasitic helminths, there is an urgent need for discovery of novel broad-spectrum anthelmintics displaying no or minimal toxicity towards the host. Silver being used since centuries for therapeutic purposes and considered safe for human consumption, we investigated anthelmintic activity of a colloidal nanosilver formulation Silversol®. Anthelmintic efficacy of the test formulation was assayed employing the nematode *Caenorhabditis elegans* as a model worm through a live-dead count.

**Results:**

Silversol® exerted anthelmintic action superior to one of the positive controls (Benzimidazole), and almost at par to another positive control (Ivermectin). At concentrations ≥ 2 ppm, it could kill all the worms present in the experimental well. Lower concentrations of silver were found to have a cuticle-damaging action on worms. Further investigation is warranted to assess whether Silversol® can exert similar potent activity against different species of parasitic helminths, and elucidate the underlying molecular mechanisms of action.

**Supplementary Information:**

The online version contains supplementary material available at 10.1186/s13104-023-06392-1.

## Introduction

Parasitic nematodes impose considerable infection burden on human and other animals, and crop production. Approximately one-third of the world population (i.e. >2 billion people) has been estimated by the WHO (World Health Organization) to be infected with one or more helminth species [1]. Many of the currently available anthelmintic drugs are experiencing a reduction in their utility owing to development of resistance among nematodes. Serious concerns have also been raised about the environmental impact of the nematicides being used for crop protection [2]. Resistance to anthelmintics in nematodes is becoming widespread, as only a few chemically dissimilar groups of anthelmintics were introduced over the past several decades. Owing to overlapping modes of action, resistance to one particular compound is likely to be accompanied by resistance to other members of the group. Multidrug resistance in cattle nematodes has been documented on farms in the Americas, New Zealand, as well as, Europe. Reports of ivermectin resistance from areas where benzimidazole resistance was already widespread are alarming [3].

Since the burden of nematode infections in humans, animals and plants is not negligible, the need for concerted effort for discovery of novel anthelmintic compounds is quite clear. Though *Caenorhabditis elegans* is not a parasitic nematode, it has been considered a useful platform for anthelmintic and nematicide discovery [4]. One of the major challenges hampering the anthelmintic drug discovery programmes is the biochemical similarity between host and parasite genomes. An ideal anthelmintic drug would be toxic to the parasites at concentrations well tolerated by the host. Silver is being used as a part of traditional medicine since long, and can be believed to be safe as a therapeutic agent. This study aimed at investigating the anti-nematode activity of a colloidal nano-silver formulation (Silversol®) employing the *C. elegans* as a model worm. It is a colloidal silver preparation reported to possess multiple biological activities. This formulation has been reported to possess microbicidal activity [5], and found application in the areas of wound healing [6], burn treatment [7], and oral care [8]. Different Silversol® products have received various regulatory approvals from competent authorities in the US, India and Canada, whose details can be accessed in [7].

## Materials and methods

### Test formulation

Silversol®: The test formulation SilverSol® (32 ppm) originally developed by American Biotech Labs (USA) was procured from Viridis BioPharma Pvt Ltd, Mumbai, India. The elemental form of zero-valent metallic silver particles contained in this product is coated with silver oxide, and the particle size ranges between 5 and 50 nm [9].

### Test organism

Wild type (N2 Bristol; CGC, University of Minnesota) *Caenorhabditis elegans* was employed as a model nematode in this study. This worm was maintained on NGM agar plates (Nematode Growing Medium :NGM; 3 g/L NaCl, 2.5 g/L peptone, 1 M CaCl_2_, 1 M MgSO_4_, 5 mg/mL cholesterol, 1 M phosphate buffer of pH 6, 17 g/L agar-agar) seeded with *E. coli* OP50 at 22 °C. For synchronization of the worm population [10], adult worms from a 4–5 days old NGM plate were first washed with sterile distilled water, and then treated with 1 mL of bleaching solution [4% Sodium hypochlorite (Merck 61,842,010,001,730) + 1 N Sodium hydroxide (HiMedia MB095-100G) + water in 1:1:3 proportion], followed by centrifugation (at 1500 rpm at 22 °C) for 1 min. Eggs in the resultant pellet were washed multiple times with sterile distilled water, and then transferred onto a new NGM plate seeded with *E. coli* OP50 (LabTIE International, Netherlands). L3-L4 stage worms appearing on this plate after 2–3 days of incubation at 22 °C were used for the anti-nematode assay and were kept on NGM plates (not seeded with *E. coli* OP50) for two days, before being challenged with silver.

### Assay for anti-nematode activity

Gnotobiotic worms obtained as described above were picked from the NGM agar plate, and were distributed into different wells of a 24-well plate (HiMedia) containing M9 buffer. Ten (hermaphrodite) worms were delivered per well. This was followed by addition of required volume (1-156 µL) of Silversol® solution. Total volume in each well was kept 1 mL. Silversol® was tested over a concentration range of 0.03-5 ppm. Three replicates for each concentration were set. Control wells contained worms in M9 buffer (with no silver). Benzimidazole (HiMedia) and ivermectin (SRL) were used as positive controls. Since these compounds were dissolved in DMSO (Merck), an appropriate vehicle control (i.e. worms in M9 buffer + 0.5%v/v DMSO) was also set. These 24-well plates were incubated at 22 °C for 5-days, and a live-dead count was performed under microscope (4X) on daily basis. Non-moving straight worms were considered as dead. Plates were tapped to confirm absence of movement in apparently dead worms. On last day of experiment, when plates could be opened, dead-looking worms were touched gently with a straight wire to further confirm lack of response.

### Live-dead staining of worms

Live-dead staining of the worms was done as described in [11, 12]. Control or Ag-exposed worms from the 24-well assay plate were transferred after 3-days of incubation into a sterile centrifuge tube (15 mL), and mixed with 1 mL of Trypan Blue (CDH Fine Chemicals; 0.4 mg/mL in distilled water) or Neutral Red (Nice Chemicals; 20 mg/mL in distilled water), and kept at 22ºC for 20 min and 2 h respectively. Then the worms were centrifuged at 700 rpm for 1 min, followed by washing with sterile M9 buffer twice. These worms were then used for microscopic observation.

### Statistics

All values reported are means of three independent experiments, and measurements are reported as mean ± standard deviation (SD). Statistical significance of the data was evaluated by applying t-test using Microsoft Excel®. *p* values ≤ 0.05 were considered to be statistically significant.

## Results and discussion

**Fig. 1:**
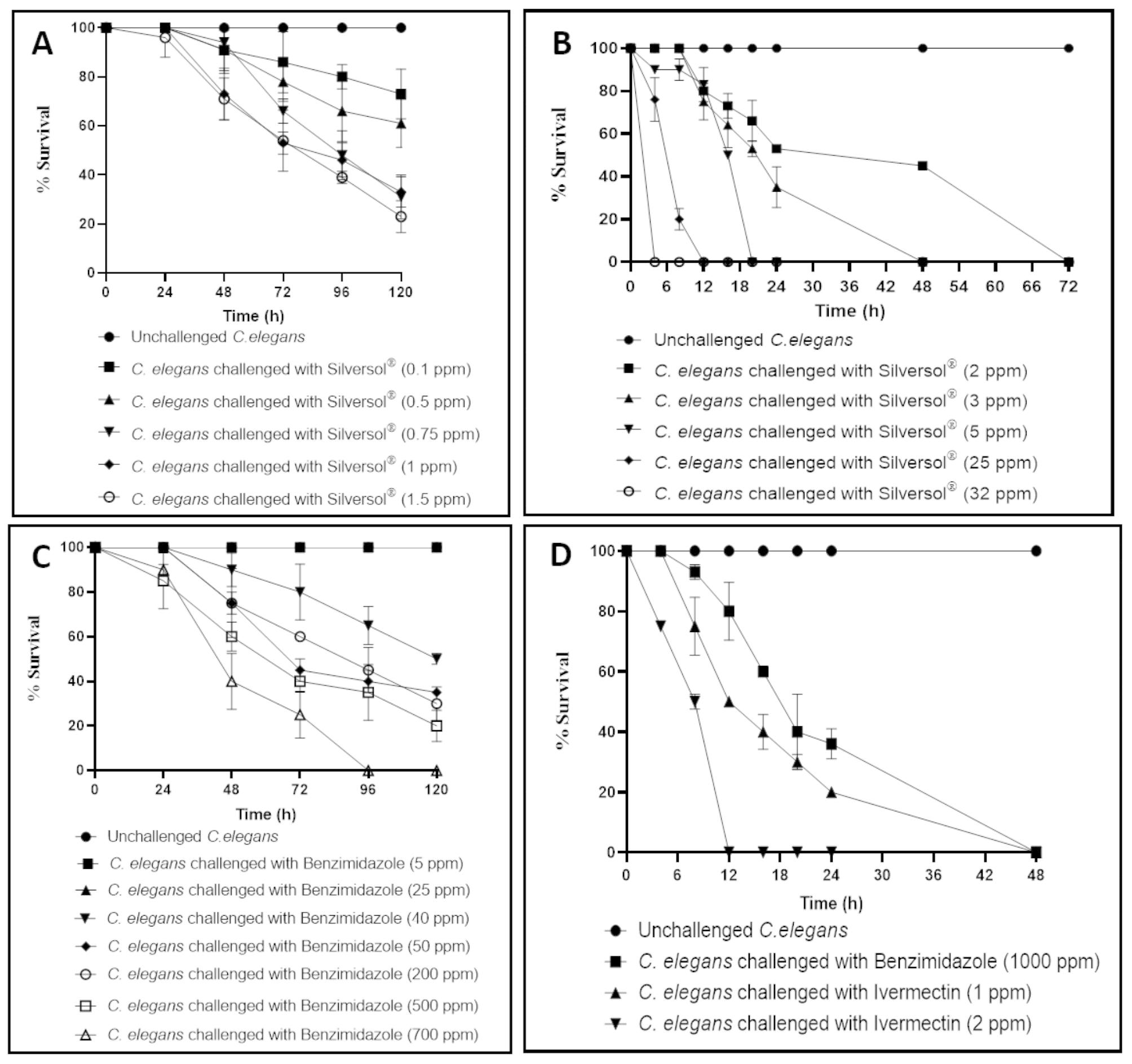
**Anthelmintic activity of Silversol**^®^**and positive control compounds.** (**A**) Silversol^®^ at concentrations 0.1–1.5 ppm could kill 27–77% worms by fifth day. Lower concentrations 0.03–0.07 ppm had no killing effect, and hence has not been shown in graph. (**B**) Silversol^®^ at ≥ 2 ppm could kill all the worms in a dose-dependent fashion. (**C**)-(**D**): Benzimidazole could kill 100% worm population only at concentrations ≥ 700 ppm. Ivermectin could do so at much lower concentrations. Since both these compounds were dissolved in DMSO, effect of DMSO (0.5%v/v) on worm survival was also investigated. DMSO at this dose did not have any effect on worm survival. Raw data pertaining to results shown in this figure is submitted as supplementary file S1.

Silversol® till 0.07 ppm did not exert any toxicity towards the worm population. Thereafter its toxicity towards the worm appeared to follow a linear dose response curve (Fig. 1A-B). Silversol® concentrations ≥ 2 ppm could kill 100% of worm population, wherein the time required for complete killing decreased with increase in concentration. While 5 ppm of benzimidazole (positive control) could not kill any worms till 120 h (Fig. 1C), same concentration of Silversol® killed all the worms in less than 24 h. Cent percent killing of worms could be achieved with benzimidazole only when the test concentration approached 1000 ppm, and it took 2 days for it to do so (Fig. 1D). Ivermectin (1 ppm) could kill 100% worms in 2 days. Its 2 ppm concentration could do so within 12 h. Thus, we found silver to be more potent anthelmintic agent than benzimidazole, but less potent than ivermectin in terms of pace of killing (Table 1). Though ivermectin is the most potent among all anthelmintic agents prescribed today for treating parasitic infections, resistance against it is also being reported [13, 14], and need for novel anthelmintic agents remains live.

**Table 1:** Comparative summary of anthelmintic activity of Silversol®

Anthelmintic agent	Concentration (ppm)	Time required for initiation of worm death (h)	Time required for ~ 50% killing of worm population (h)	Time required for cent % killing of worm population (h)
Silversol®	0.1	48	Maximum killing observed till fifth day was < 50%	
	0.5	48		
	0.75	48	96	Maximum killing observed till fifth day was < 100%
	1	48	72	
	1.5	24	72	
	2	12	24	72
	3	12	20	48
	5	4	Not determined	20
	25	4	< 8	12
	32	2	Not determined	4
Benzimidazole (HiMedia)	40	48	120	Maximum killing observed till fifth day was < 100%
	50	48	72	
	200	48	96	
	500	24	72	
	700	24	48	96
	1000	8	20	48
Ivermectin (SRL)	1	8	12	48
	2	4	8	12

**Fig. 2:**
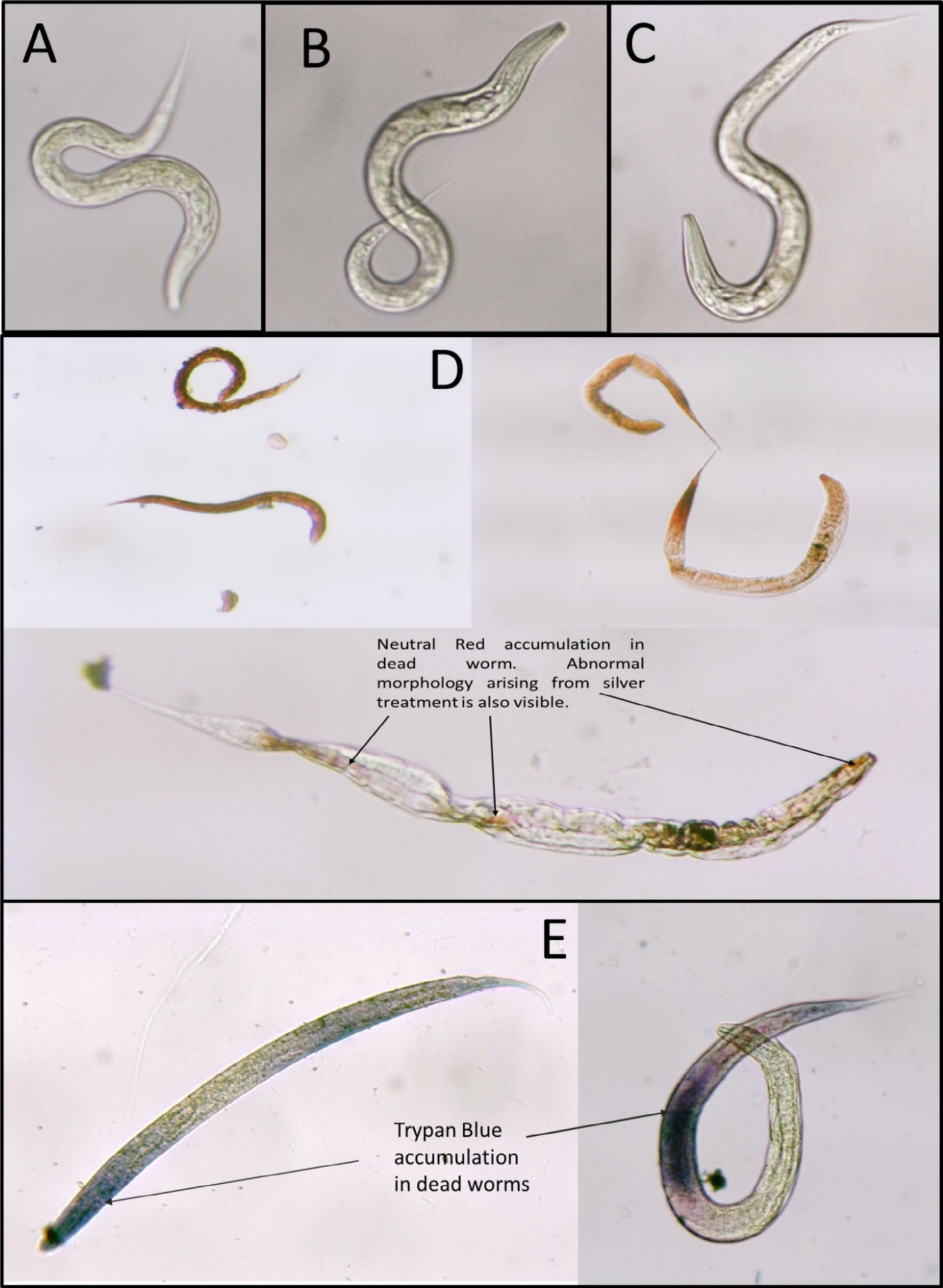
**Uptake of dyes by dead, but not the live worms. A.** Unstained live worm; **B**. Live worm kept with neutral red, but not getting stained; **C.** Live worm kept with trypan blue, but not getting stained; **D**. Silver-exposed dead worms getting stained with neutral red; **E**. Silver-exposed dead worms getting stained with trypan blue. Videos (F-H and J-L) recording these experiments are available at: 10.17605/OSF.IO/S9BMZ. Images and videos were captured using Magnus Camera (5.1 MP) attached to Magnus MLX-B Plus microscope (10X objective)

Since live-dead microscopic count is susceptible to observer bias up to some extent, it may be considered a semi-quantitative method. As an additional confirmation of the reliability of our live-dead count, we subjected the control (live) as well as Ag-exposed (dead) worms to staining by two different dyes, Neutral Red or Trypan Blue. Intact cuticle of the live and healthy worm prevents entry of the stains into the worm body, while cuticle of the dead worm being more permeable allows partial entry of the dye into the worm, and hence only the dead worms appear stained (Fig. 2). Suitability of Neutral Red staining for detection of anthelmintic activity mediated by cuticular damage in nematodes has earlier been reported by Phiri et al. [11].

**Fig. 3:**
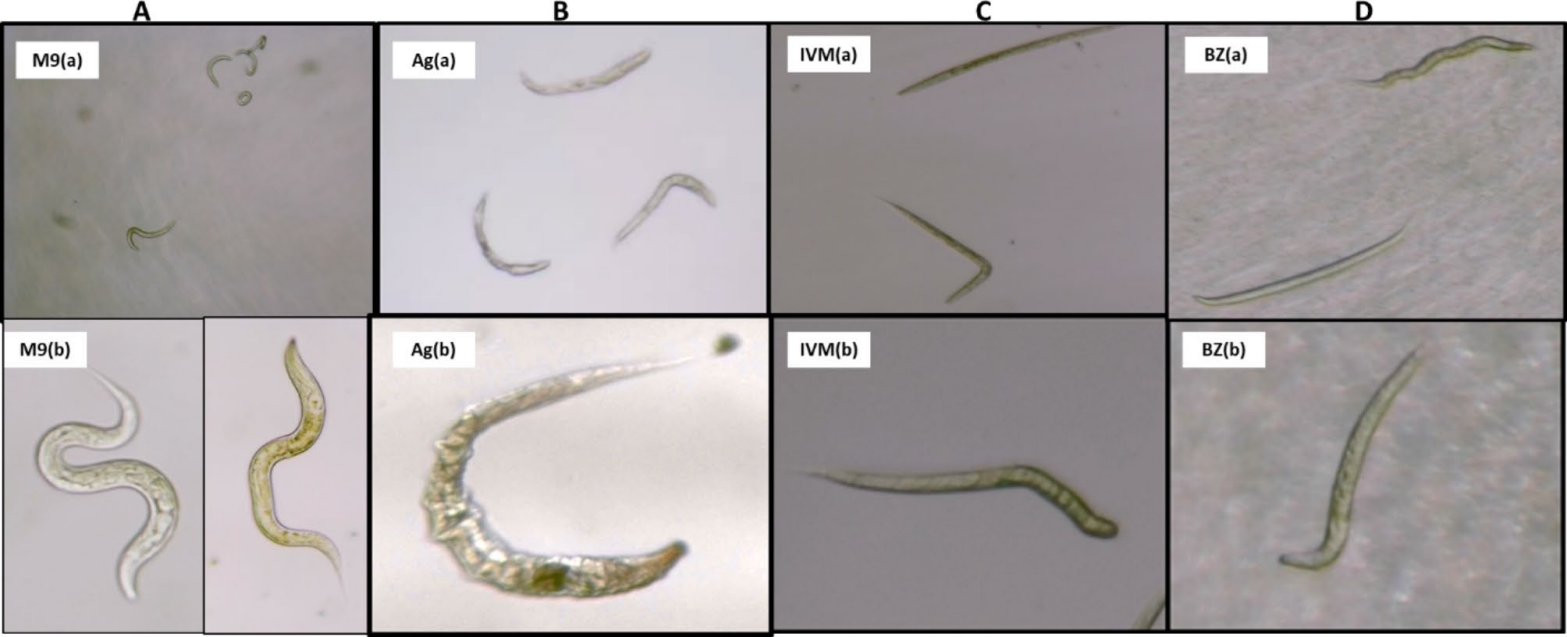
Images demonstrating healthy control worms (Panel **A**); silver-induced cuticle damage in worms (Panel **B**); and ivermectin- or benzimidazole- induced paralysis leading to worm death (Panel **C**-**D**). Each panel contains two images, each representing different microscopic fields of the same well; lower panel has been included to show a single worm for better observation. Videos (A, C-E, and I) recording these experiments are available at: 10.17605/OSF.IO/S9BMZ. Images and videos were captured using Magnus Camera (5.1 MP) attached to Magnus MLX-B Plus microscope (4X or 10X objective). Images M9(b) and Ag(b) were captured at 10X M9: control (72 h); Ag: Silversol^®^ (1 ppm; 72 h); IVM: Ivermectin (1 ppm; 12 h); BZ: Benzimidazole (1000 ppm; 20 h)

Mode of action of silver seems to be different from that of benzimidazole and ivermectin as latter two agents induced paralysis prior to death unlike silver. Impairment of locomotion in *C. elegans* has already been known among multiple effects of benzimidazoles on this worm. Similarly elicitation of a potent and persistent paralysis of nematode pharyngeal and body wall musculature due to ivermectin is also known [15]. Silver-treated worms appeared with damaged cuticle (Figs. 2 and 3; Supplementary Videos: 10.17605/OSF.IO/S9BMZ). However, cuticle damage could be observed only with lower concentrations of silver, and not with higher concentrations. Higher silver concentrations may be exerting their effect in a different fashion (i.e. not necessarily causing cuticle damage). Pimentel-Acosta et al. [16] also reported that silver nanoparticles at high and low concentrations activated dysregulation of similar biological processes (detoxification, neurotoxicity, modulation of cell signaling, embryonic development, reproduction, and tegument organization) by different mechanisms in metazoan parasites of fish. Since cuticle can act as a diffusion barrier for various anthelmintic compounds [17], combination of silver with conventional anthelmintic agents may allow better penetration of worm body. Silver nano-particles were shown to pass cell membranes and getting internalized in *C. elegans* by Meyer et al. [18], and they proposed part of the toxicity observed to be mediated by ionic silver. Perhaps the potency of silver nanoparticles is also a function of method of their synthesis, as silver nanoparticles synthesized by different methods have been reported to be effective against *Meloidogyne* species at widely different concentrations [19, 20].

Though avermectins (including ivermectin) are the most widely used anti-parasitic drugs, concerns have been raised regarding their safety in the animals. Harmful effects (nephrotoxicity, hepatotoxicity, reproductive toxicity, neurotoxicity, endocrine disruption, etc.) of avermectins are rooted in their targeting GABA and glutamate-gated chloride channels, which are present both in the parasites as well as the host animals [21]. Toxicity of currently used anthelmintics like 3-[2-(1 H-Benzimidazol-2-ylsulfanyl)-ethyl]-1,3-oxazolidin-2-one (a benzimidazole derivative), levamisole and avermectin, has been reported in rat [22], sheep [23], and human system [24] respectively. Silversol^®^ has been shown to be safe for human consumption [7], and silver in general up to 5 µg/Kg body weight per day is believed to be safe for humans (https://www.govinfo.gov/content/pkg/FR-1999-08-17/html/99-21253.htm). While considerable insight has been developed regarding the anti-nematode mode of action of albendazoles and avermectins [25], that is not the case with silver though its anthelmintic action has been known [26]. Further investigation on gene expression profile of silver-treated worms can elucidate novel targets and modes of action, and these targets may be useful in discovery of more new anthelmintics.

## Electronic supplementary material

Below is the link to the electronic supplementary material.

Table S1: Survival of worms challenged with different anthelmintic agents
Video content supplemental to Figure 2-3 can be viewed at: https://doi.org/10.17605/OSF.IO/S9BMZ

## Data Availability

All the data has been provided within main manuscript or supplementary files.

## Declarations

### Limitations of the study

Though *C. elegans* is a valid platform for preliminary investigation on anthelmintic activity of any test formulation, it is not a parasite. The parasitic helminths may or may not exert a susceptibility profile similar to that of *C. elegans*. This study did not test Silversol^®^ against any such parasitic helminth.

## Abbreviations

NGM: Nematode Growing Medium
DMSO: Dimethyl Sulphoxide
