# Supplementary material for: Potent anthelmintic activity of a colloidal nano-silver formulation (Silversol®) against the model worm Caenorhabditis elegans
Source: BMC Res Notes. 2023 Jul 3;16:130. doi: 10.1186/s13104-023-06392-1 (PMC10318827; doi:10.1186/s13104-023-06392-1)
Supplement: Supplementary file 1 — Table S1: Survival of worms challenged with different anthelmintic agents Video content supplemental to Figure 2-3 can be viewed at: https://doi.org/10.17605/OSF.IO/S9BMZ [file 13104_2023_6392_MOESM1_ESM.docx]

Potent anthelmintic activity of a colloidal nano-silver formulation (Silversol^®^) against the model worm *Caenorhabditis elegans*

Gemini Gajera^1^, Chhaya Godse^2^, Anselm DeSouza^2^, Dilip Mehta^2^, Vijay Kothari^1*^

^1^Institute of Science, Nirma University, Ahmedabad- 382481, India;

^2^Viridis BioPharma Pvt. Ltd., Mumbai, India

^*^Correspondence: vijay.kothari@nirmauni.ac.in

**Table S1: Survival of worms challenged with different anthelmintic agents**

|  |  | **Silversol** | | | | | | | | | |
| --- | --- | --- | --- | --- | --- | --- | --- | --- | --- | --- | --- |
|  |  | Concentration (ppm) | | | | | | | | | |
|  |  | **0.1** | **0.5** | **0.75** | **1** | **1.5** | **2** | **3** | **5** | **25** | **32** |
| Hours | 4 | 100 | 100 | 100 | 100 | 100 | 100 | 100 | 90±0*** | 76±10.2*** | 0 |
|  | 8 | 100 | 100 | 100 | 100 | 100 | 100 | 100 | 90±5*** | 20±5** |  |
|  | 12 | 100 | 100 | 100 | 100 | 100 | 80±0*** | 75±8.5** | 83±8** | 0 |  |
|  | 16 | 100 | 100 | 100 | 100 | 100 | 73±5.8** | 64±10.5** | 50±0*** | 0 |  |
|  | 20 | 100 | 100 | 100 | 100 | 100 | 66±9.6** | 53±3.8*** | 0 | 0 |  |
|  | 24 | 100 | 100 | 100 | 100 | 96±8*** | 53±1.6** | 35±9.5** | 0 | 0 |  |
|  | 48 | 91±9.6** | 91±8.5** | 94±5*** | 73±10.5** | 71±8.6** | 45±0** | 0 | 0 | 0 |  |
|  | 72 | 86±12.5** | 78±8.0** | 66±5** | 53±4.5*** | 54±12.5*** | 0 | 0 | 0 | 0 |  |
|  | 96 | 80±5.0*** | 66±12.5** | 48±10*** | 46±7** | 39±2.5*** | 0 | 0 | 0 | 0 |  |
|  | 120 | 73±10.1** | 61±9.8* | 31±9** | 33±6.2** | 23±6.5** | 0 | 0 | 0 | 0 |  |

[Values presented here are % of surviving worms; Wherever SD is not shown, its value is zero; Worm survival in control wells (i.e. worms kept in M9buffer) and vehicle control wells (i.e. worms in M9 buffer supplemented with 0.5%v/v DMSO) was 100% till 120 h; *p≤0.05, **p≤0.01, ***p≤0.001]

|  |  | **Benzimidazole** | | | | | | | |
| --- | --- | --- | --- | --- | --- | --- | --- | --- | --- |
|  |  | Concentration (ppm) | | | | | | | |
|  |  | **5** | **25** | **40** | **50** | **200** | **500** | **700** | **1000** |
| Hours | 4 | 100 | 100 | 100 | 100 | 100 | 100 | 100 | 100 |
|  | 8 | 100 | 100 | 100 | 100 | 100 | 100 | 100 | 93±2.5*** |
|  | 12 | 100 | 100 | 100 | 100 | 100 | 100 | 100 | 80±9.6*** |
|  | 16 | 100 | 100 | 100 | 100 | 100 | 100 | 100 | 60±1*** |
|  | 20 | 100 | 100 | 100 | 100 | 100 | 100 | 100 | 40±12.5** |
|  | 24 | 100 | 100 | 100 | 100 | 100 | 85±12.5** | 90±2.5*** | 36±5** |
|  | 48 | 100 | 100 | 90±7.5** | 75±15.5** | 75±5*** | 60±6.5*** | 40±12.5** | 0 |
|  | 72 | 100 | 100 | 80±12.5*** | 45±5** | 60±0*** | 40±5** | 25±10.5*** | 0 |
|  | 96 | 100 | 100 | 65±8.6** | 40±5** | 45±10.2** | 35±12.5** | 0 | 0 |
|  | 120 | 100 | 100 | 50±2.5** | 35±1.5** | 30±7.5** | 20±7*** | 0 | 0 |

|  |  |  | **Ivermectin** | |
| --- | --- | --- | --- | --- |
|  |  |  | Concentration (ppm) | |
|  |  |  | **1** | **2** |
| Hours | 4 |  | 100 | 75±1*** |
|  | 8 |  | 75±9.6*** | 50±2.5** |
|  | 12 |  | 50±0*** | 0 |
|  | 16 |  | 40±5.8** | 0 |
|  | 20 |  | 30±2.5*** | 0 |
|  | 24 |  | 20±0*** | 0 |
|  | 48 |  | 0 | 0 |
|  | 72 |  | 0 | 0 |
|  | 96 |  | 0 | 0 |
|  | 120 |  | 0 | 0 |
